# Postoperative pulmonary complications after esophagectomy: risk factors and prediction model

**DOI:** 10.1093/dote/doag041

**Published:** 2026-04-21

**Authors:** Dillen C van der Aa, Rahaf Khatib, Wietse J Eshuis, Freek Daams, Suzanne S Gisbertz, Mark van Berge Henegouwen

**Affiliations:** Department of Surgery, Amsterdam UMC, Location University of Amsterdam, Meibergdreef 9, Amsterdam, North Holland, the Netherlands; Cancer Center Amsterdam, Cancer Treatment and Quality of Life, Amsterdam, North Holland, the Netherlands; Department of Gastroenterology and Hepatology, Amsterdam Gastroenterology Endocrinology Metabolism, Amsterdam UMC, Location University of Amsterdam, Meibergdreef 9, Amsterdam, North Holland, the Netherlands; Department of Surgery, Amsterdam UMC, Location University of Amsterdam, Meibergdreef 9, Amsterdam, North Holland, the Netherlands; Department of Surgery, Amsterdam UMC, Location University of Amsterdam, Meibergdreef 9, Amsterdam, North Holland, the Netherlands; Cancer Center Amsterdam, Cancer Treatment and Quality of Life, Amsterdam, North Holland, the Netherlands; Department of Gastroenterology and Hepatology, Amsterdam Gastroenterology Endocrinology Metabolism, Amsterdam UMC, Location University of Amsterdam, Meibergdreef 9, Amsterdam, North Holland, the Netherlands; Cancer Center Amsterdam, Cancer Treatment and Quality of Life, Amsterdam, North Holland, the Netherlands; Department of Surgery, Amsterdam UMC, Location Vrije Universiteit, De Boelelaan 1118, Amsterdam, North Holland, the Netherlands; Department of Surgery, Amsterdam UMC, Location University of Amsterdam, Meibergdreef 9, Amsterdam, North Holland, the Netherlands; Cancer Center Amsterdam, Cancer Treatment and Quality of Life, Amsterdam, North Holland, the Netherlands; Department of Gastroenterology and Hepatology, Amsterdam Gastroenterology Endocrinology Metabolism, Amsterdam UMC, Location University of Amsterdam, Meibergdreef 9, Amsterdam, North Holland, the Netherlands; Department of Surgery, Amsterdam UMC, Location University of Amsterdam, Meibergdreef 9, Amsterdam, North Holland, the Netherlands; Cancer Center Amsterdam, Cancer Treatment and Quality of Life, Amsterdam, North Holland, the Netherlands; Department of Gastroenterology and Hepatology, Amsterdam Gastroenterology Endocrinology Metabolism, Amsterdam UMC, Location University of Amsterdam, Meibergdreef 9, Amsterdam, North Holland, the Netherlands

**Keywords:** esophagectomy, prediction model, pulmonary complications, risk factors

## Abstract

**Background:**

Postoperative pulmonary complications (PPCs) affect up to one-third of patients undergoing esophagectomy and remain a major contributor to postoperative morbidity. This study aimed to identify pre- and perioperative risk factors for PPCs and to develop a predictive model.

**Methods:**

This retrospective cohort study included patients who underwent esophagectomy for esophageal or gastroesophageal junction cancer at Amsterdam UMC between 2013 and 2023. PPCs included pneumonia, pleural effusion, pneumothorax, atelectasis, respiratory failure, aspiration, acute respiratory distress syndrome, tracheobronchial fistula, and persistent air leakage. Univariable and multivariable logistic regression with backward selection were used to identify predictors. Model performance was assessed with the area under the receiver operating characteristic curve (AUC). Statistical significance was set at *P* < 0.05.

**Results:**

Among 960 patients, 254 (26.5%) developed at least one PPC. Independent predictors were smoking status (former: OR 1.45, 95% CI 1.03–2.06; current: OR 1.74, 95% CI 1.15–2.63), non-epidural analgesia (paravertebral: OR 1.50, 95% CI 1.03–2.17; other: OR 1.57, 95% CI 0.88–2.82), and cervical versus intrathoracic anastomosis (OR 1.64, 95% CI 1.17–2.29). Drain configuration also influenced the risk of PPC: one-sided double drains were protective (OR 0.48, 95% CI 0.25–0.91), whereas bilateral drains increased the risk (OR 2.60, 95% CI 1.38–4.87), compared with one-sided single drains. The model demonstrated modest discrimination after validation of AUC: 0.598.

**Conclusion:**

Smoking, paravertebral analgesia, cervical anastomosis, and bilateral drains were independently associated with increased PPC risk. Although predictive performance was modest, these modifiable and structural factors inform perioperative management. Future models should incorporate intraoperative and physiological variables to improve risk stratification.

## INTRODUCTION

Esophageal cancer ranks as the 11th most common malignancy worldwide and is the 7th leading cause of cancer-related mortality.^1^ Curative-intent treatment typically involves multimodal therapy, consisting of neoadjuvant chemoradiotherapy or perioperative chemotherapy followed by esophagectomy.^2^^,^^3^ Minimally invasive esophagectomy (MIE) has increasingly replaced open esophagectomy as the preferred approach in high-volume centers, owing to its association with reduced morbidity, shorter length of hospital stay, and lower rates of pulmonary complications.^4–6^

Nevertheless, postoperative pulmonary complications (PPCs) remain among the most frequent and clinically significant adverse events following esophagectomy, affecting up to 30% of patients.^7–9^ PPCs, including pneumonia (the most frequent), respiratory failure, pleural effusion, empyema, and pneumothorax, are associated with increased postoperative mortality, prolonged hospitalization, and impaired long-term survival.^10–12^

Given their impact on clinical outcomes and resource utilization, PPCs represent a key target for perioperative optimization. Accurate identification of risk factors may guide patient selection, inform prehabilitation strategies, and support individualized perioperative planning. Established known risk factors include impaired preoperative pulmonary function,^13^^,^^14^ a history of smoking, surgical approach, and postoperative serum albumin levels.^7^^,^^15^

Enhanced risk stratification may inform surgical decision-making, optimize patient selection, and support tailored perioperative management strategies.^9^ Although various predictive models have been proposed to assist in treatment planning and risk assessment, majority require further refinement and external validation.^16–18^

Therefore, the aim of this study was to identify relevant pre- and perioperative risk factors for PPCs in patients undergoing esophagectomy for esophageal cancer, and to develop a multivariable risk model to support clinical decision-making.

## METHODS

### Study design

The dataset used for this single-center retrospective study was prospectively maintained within Department of Surgery at the Amsterdam UMC, Netherlands. The dataset consists of 1002 patients that underwent esophagectomy from January 2013 to December 2023.

### Patient population

Eligible patients were ≥ 18 years, diagnosed with esophageal or gastroesophageal junction carcinoma (clinical stage T1–4b, N1–3, M0).

### Outcome measures

The primary outcome was the occurrence of postoperative pulmonary complications. Secondary independent variables included identification of clinical risk and protective factors for PPCs.

### Surgery

The surgical techniques at Amsterdam UMC, including transthoracic and historical transhiatal esophagectomy as well as both intrathoracic and cervical anastomoses, have been described previously.^19^ All transhiatal procedures were performed with a cervical anastomosis. The current standard is a minimally invasive Ivor Lewis procedure. Cervical anastomoses were selectively performed following multidisciplinary discussion, typically for mid- or proximal esophageal tumors, high mediastinal lymph node involvement, or when neoadjuvant radiotherapy fields extended into the superior mediastinum.

### Lymphadenectomy

In our center, lymphadenectomy during transthoracic esophagectomy is performed according to a standardized protocol. During the abdominal phase (laparoscopic or robotic), a routine upper abdominal lymph node dissection is carried out (TIGER stations 14–19).^20^

For intrathoracic anastomosis, mediastinal lymphadenectomy typically includes stations 8, 9, 11, 12, and 13. In cases of cervical anastomosis, the thoracic phase is performed first, with a more extended mediastinal dissection (stations 4, 5, 8–13), followed by the abdominal phase as described above. When clinically indicated, a cervical lymph node dissection (station 2) is added.

### Analgesia

Postoperative analgesia was provided using either thoracic epidural or paravertebral catheter-based regimens.^21^ Epidural catheters were placed preoperatively and infused continuously with bupivacaine and sufentanil. Paravertebral catheters were inserted intraoperatively under thoracoscopic guidance and infused with bupivacaine. In both groups, additional analgesia was administered as needed, and catheters were typically removed after 3 days.

### Drains

In thoracoscopic and robotic esophagectomy, a chest tube was routinely placed in the right pleural cavity at the end of the thoracic phase. From January 2021 onward, an additional Jackson-Pratt (JP) drain was positioned on the right side to allow fluid monitoring, enabling early removal of the chest tube (postoperative day 1) if the lung was fully expanded on chest X-ray and no air leakage was observed.^22^ In contrast, patients undergoing open esophagectomy received a chest tube on each side for uni- or bilateral pleural drainage.

**Fig. 1:**
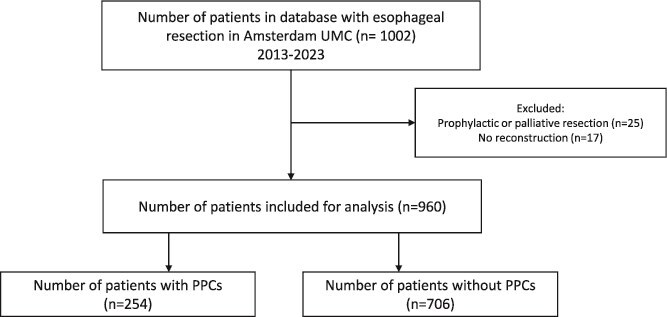
Flow chart of selection procedure of included patients in analysis.

### Definitions

Postoperative pulmonary complications (PPCs) were defined according to the international consensus of the Esophagectomy Complications Consensus Group (ECCG).^22^^,^^23^ PPCs included pneumonia, pleural effusion requiring drainage, pneumothorax requiring intervention, atelectasis requiring bronchoscopy, respiratory failure requiring reintubation, acute aspiration, acute respiratory distress syndrome (ARDS; Berlin definition^24^), tracheobronchial injury, and persistent air leakage necessitating chest tube maintenance for ˃10 days (see Supplementary Table 1 for detailed definitions). All PPCs were assessed during index admission and before discharge.

### Independent variables

Patient characteristics included age (continuous, calculated from date of birth to surgery), sex (male/female), body mass index (BMI) (<30/≥ 30), smoking status (non-smoker, former smoker, current smoker), diabetes mellitus (yes/no), and chronic lung disease (COPD or asthma, yes/no). Preoperative pulmonary function was assessed by FEV1/VC ratio (>70% vs. ≤70%). Tumor histology was categorized as adenocarcinoma, squamous cell carcinoma, or other (e.g. signet ring cell, poorly differentiated, neuroendocrine, or neuro-squamous carcinoma). Additional variables included ASA classification (I, II, III/IV), use of salvage therapy (yes/no), and neoadjuvant treatment (none, chemotherapy, chemoradiotherapy).

Surgical variables included analgesia type (epidural, paravertebral, or other—e.g. sufentanil pump/esketamine (IV pump), drain configuration (one-sided, one-sided + JP drain, or bilateral), type of resection (transthoracic/transhiatal), surgical approach (open, hybrid, minimally invasive esophagectomy), and location of anastomosis (intrathoracic or cervical). The other surgical protocols have been described previously.^19^

### Statistical analysis

Analyses were performed using SPSS (v28), R (v4.2.2), and RStudio (v2024.12.0). Using the *pmsampsize* package, we calculated the required sample size based on a 27% event rate, a C-index of 0.7, a shrinkage factor of 0.8, and 16 variables (25 degrees of freedom). This yielded a target sample size of 965 to minimize the risk of overfitting. Missing FEV1/VC values (8.3%) were deemed missing at random and imputed using fully conditional specification (FCS) with nine imputations, including all independent variables and no auxiliary variables. Results were pooled using Rubin’s rules.^25^

Multivariable logistic regression with backward selection was performed on the imputed datasets to identify predictors of PPCs (dichotomous outcome). Internal validation was done via bootstrapping and multiple imputation in RStudio.^26^ Univariable logistic regression was conducted separately in SPSS. Linearity assumptions were assessed for continuous variables; BMI failed this assumption and was therefore dichotomized. Statistical significance was set at *P* < 0.05.

To account for potential temporal changes in surgical practice and postoperative management, we performed a sensitivity analysis testing for an interaction between chest drain strategy and date of surgery.

## RESULTS

One thousand and two patients underwent esophageal resection between 2013 and 2023. After exclusion of patients due to palliative resection or no reconstruction, 960 patients were included in the analysis (Fig. 1). Of these, 254 patients (26.5%) developed a PPC (Fig. 1).

No significant differences were observed in baseline characteristics between patients with and without a PPC in age, sex, or BMI. The median age in the total cohort was 66 years [21–89], and 76.9% (n = 738) were male. Adenocarcinoma was the most common histology with 79%. The BMI distribution showed a 5.86:1 ratio for <30 versus ≥30. A total of 62 patients (24.4%) in the PPC+ group were current smokers. An FEV1/VC ratio ≤ 70% was observed in 36.5% of patients. ASA III/IV classification was present in 23.5% of the cohort (Table 1).

**Table 1:** Baseline characteristics

	**Postoperative pulmonary complication (+) N = 254 (%)**	**Postoperative pulmonary complication (−) N = 706 (%)**
Factor		
**Patient characteristics**		
**Sex**		
Male	188 (74)	550 (77.9)
Female	66 (26)	156 (22.1)
**Age** (Mean, SD) (in years)	64.8 (8.8)	64.8 (8.8)
**BMI** (Mean, SD)	26 (4.9)	25.5 (4.1)
**BMI** (in kg/m^2^)		
< 30	209 (82.3)	611 (86.5)
≥ 30	45 (17.7)	95 (13.5)
**FEV1VC** ^**a**^		
>70	152 (59.8)	432 (61.2)
≤70	102 (40.2)	274 (38.8)
**ASA-score**		
I	45 (17.7)	128 (18)
II	143 (56.3)	418 (59)
III/IV	66 (26)	160 (23)
**Smoking**		
Non-smoker	70 (27.6)	250 (35.4)
Former smoker	122 (48)	327 (46.3)
Smoker	62 (24.4)	129 (18.3)
**Diabetes mellitus**		
No	219 (86.2)	629 (89)
Yes	35 (13.8)	77 (11)
**Chronic lung disease**		
No	227 (89.4)	656 (92.9)
Yes	27 (10.6)	50 (7.1)
**Treatment characteristics**		
**Neo-adjuvant treatment**		
No neo-adjuvant treatment	25 (9.8)	46 (6.5)
Neo-adjuvant chemoradiation	214 (84.3)	604 (85.6)
Neo-adjuvant chemotherapy	15 (5.9)	56 (7.9)
**Histological type**		
Adenocarcinoma	188 (74)	574 (81)
Squamous cell carcinoma	60 (23.6)	113 (16)
Other^b^	6 (2.4)	19 (3)
**Approach surgery**		
Open	12 (4.7)	22 (3.1)
Hybrid	28 (11)	58 (8.2)
MIE	214 (84.3)	626 (88.7)
**Salvage**		
No	241 (94.9)	686 (97.2)
Yes	13 (5.1)	20 (2.8)
**Drain type**		
One-sided	218 (85.8)	619 (87.7)
One-sided + JP drain	12 (4.7)	65 (9.2)
Bilateral	24 (9.5)	22 (3.1)
**Location of anastomosis**		
Intrathoracic	169 (66.5)	548 (77.6)
Cervical	85 (33.5)	158 (22.4)
**Analgesia type**		
Epidural	177 (69.7)	543 (76.9)
Paravertebral	56 (22)	127 (18)
Other^c^	21 (8.3)	36 (5.1)
**Type resection**		
Transhiatal	12 (4.7)	34 (4.8)
Transthoracic	242 (95.3)	672 (95.2)

^a^Variable with missing data.

^b^Signet ring cell-, poorly differentiated-, neuroendocrine, neuro-squamous carcinoma.

^c^Medication (such as: sufentanil pump and/or esketamine).

Abbreviations: PPC, postoperative pulmonary complication; BMI, body mass index; FEV1, Forced expiratory volume in 1 second; ASA-score, American society of Anesthesiologists Physical status; COPD, chronic obstructive pulmonary disease; MIE, minimally invasive esophagectomy (MIE Thoracic/MIE Abdominal); JP, Jackson-Pratt.

There were no statistically significant differences in pathological outcomes between PPC+ and PPC-groups, including tumor stage, nodal involvement. Patients who experienced a pulmonary complication more often had additional non-pulmonary complications compared to those without pulmonary complications (Table 2).

**Table 2:** Postoperative complications and pathology

	**Postoperative pulmonary complication (+) N = 254 (%)**	**Postoperative pulmonary complication (−) N = 706 (%)**
**Postoperative complication**	254 (100)	317 (45.0)
**Pulmonary complications**		
Pneumonia	126 (49.6)	0
Pneumonia CD ≥ 3	18 (7.1)	0
Pleura effusion	80 (31.5)	0
Pneumothorax	45 (17.7)	0
Respiratory failure	24 (9.4)	0
Atelectasis	11 (4.3)	0
Prolonged intubation (>24 h)	9 (3.5)	0
Acute aspiration	4 (1.6)	0
ARDS	5 (2.0)	0
Tracheobronchial fistula	3 (1.2)	0
**Cardiac complications**	86 (33.9)	3 (1.2)
**Anastomotic leakage**	68 (26.8)	59 (8.4)
Grade 1	4 (1.6)	5 (0.7)
Grade 2	40 (15.7)	44 (6.2)
Grade 3	24 (9.4)	10 (1.4)
**Urological complications**	17 (6.7)	22 (3.1)
**Recurrent laryngeal nerve complications**	11 (4.3)	9 (1.3)
**Chyle leakage**	33 (13.0)	31(4.4)
**Other complication(s)**	49 (19.3)	71 (10.1)
**Pathology**		
**(y)p P-stage**		
0	60 (23.6)	162 (22.9)
1	49 (19.3)	139 (19.7)
2	39 (15.4)	122 (17.3)
3	101 (39.8)	268 (37.9)
4	5 (19.7)	15 (2.1)
**(y)p N-stage**		
0	145 (57.1)	403 (57.1)
1	56 (22.0)	161 (22.8)
2	32 (12.6)	83 (11.8)
3	21 (8.3)	59 (8.3)

Abbreviations: PPCs, postoperative pulmonary complications; CD, Clavien–Dindo classification; ARDS, acute respiratory distress syndrome (Berlin definition); (y)p, pathological stage after neoadjuvant therapy (if given), according to the 8th edition AJCC/UICC TNM classification.

The median duration of hospital stay was 10 days. Univariable analysis showed significantly increased odds for PPCs in patients with squamous cell carcinoma (OR 1.621, 95% CI 1.138–2.309, *P* = 0.007), current smoking status (OR 1.723, 95% CI 1.152–2.577, *P* = 0.008), and bilateral drain placement (OR 3.098, 95% CI 1.702–5.637, *P* < 0.001). Cervical anastomosis (OR 1.744, 95% CI 1.273–2.390, *P* < 0.001) was associated with increased risk compared to intrathoracic anastomosis (Table 3).

**Table 3:** Risk factors for postoperative pulmonary complications

	**Univariable Analysis**		
**Variable**	**OR**	**95% CI**	***P*-value**
**Sex**			
Male	Ref		
Female	1.238	(0.888–1.725)	0.208
**Age** (in years)	1.000	(0.984–1.016)	0.989
**BMI** (in Kg/m^2^)			
< 30	Ref		
≥ 30	1.385	(0.940–2.041)	0.100
**FEV1VC** (in %)			
>70			
≤70	1.083	(0.799–1.466)	0.608
**ASA-score**			
I	Ref		
II	0.973	(0.659–1.436)	0.891
≥III	1.173	(0.752–1.830)	0.481
**Smoking**			
Non smoker	Ref		
Former smoker	1.342	(0.958–1.879)	0.087
Smoker	1.723	(1.152–2.577)	**0.008**
**Diabetes mellitus**			
No	Ref		
Yes	1.306	(0.851–2.003)	0.222
**Chronic lung disease (COPD/Asthma)**			
No	Ref		
Yes	1.561	(0.954–2.552)	0.076
**Neo-adjuvant therapy**			
No neoadjuvant therapy	Ref		
Chemotherapy	0.493	(0.233–1.043)	0.064
Chemoradiotherapy	0.652	(0.391–1.1087)	0.101
**Histology**			
Adenocarcinoma	Ref		
Squamous cell carcinoma	1.621	(1.138–2.309)	**0.007**
Other histology^a^	0.964	(0.379–2.450)	0.939
**Approach surgery**			
Open	Ref		
Hybrid	0.942	(0.410–2.163)	0.887
MIE	0.655	(0.321–1.339)	**0.246**
**Salvage**			
No	Ref		
Yes	1.850	(0.906–3.777)	0.091
**Drain type**			
One-sided (right or left)	Ref		
One-sided + JP (right or left + JP)	0.524	(0.278–0.989)	**0.046**
Bilateral	3.098	(1.702–5.637)	**<0.001**
**Drain type** ^b^			
Double- sided	Ref		
One-sided	0.323	(0.177–0.588)	**<0.001**
One-sided double	0.169	(0.073–0.394)	**<0.001**
**Location of anastomosis**			
Intrathoracic	Ref		
Cervical	1.744	(1.273–2.390)	**<0.001**
**Analgesia type**			
Epidural	Ref		
Paravertebral	1.308	(0.914–1.873)	0.142
Other^c^	1.776	(1.010–3.123)	**0.046**
**Type resection**			
Transhiatal	Ref		
Transthoracic	1.020	(0.520–2.003)	0.953

^a^Signet ring cell-, poorly differentiated-, neuroendocrine, neuro-squamous carcinoma.

^b^Different reference category.

^c^Medication (such as: sufentanil pump and/or esketamine).

Abbreviations: FEV1, Forced expiratory volume in 1 second; BMI, body mass index; ASA-score, American society of Anesthesiologists Physical status; COPD, chronic obstructive pulmonary disease; MIE, minimally invasive esophagectomy (MIE Thoracic/MIE Abdominal); JP, Jackson-Pratt.

**Fig. 2:**
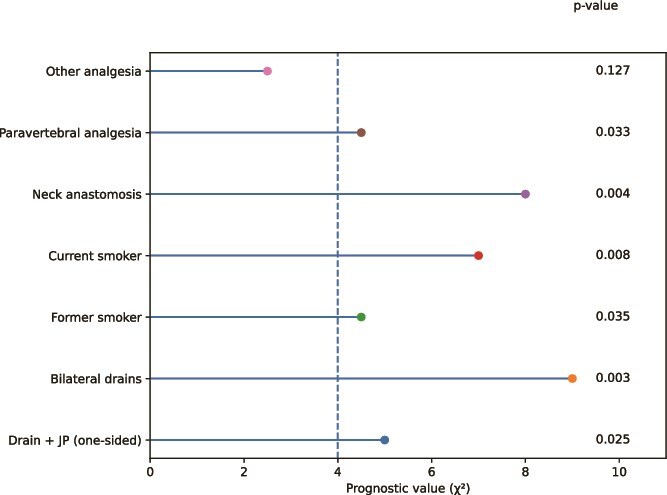
Forest plot of predictors of postoperative pulmonary complications.

Multivariable logistic regression identified four independent predictors of PPCs (Table 4, Fig. 2). Smoking status was strongly associated with risk: former smokers (OR 1.43, 95% CI 1.01–2.03) and current smokers (OR 1.73, 95% CI 1.14–2.61) both had higher odds compared with non-smokers. Analgesia type also influenced PPCs: paravertebral analgesia increased risk relative to epidural (OR 1.50, 95% CI 1.03–2.18), while other regimens (e.g. sufentanil or esketamine-based) showed a similar, though non-significant, trend (OR 1.53, 95% CI 0.85–2.75). Anastomotic location was significant, with cervical anastomosis carrying higher odds than intrathoracic (OR 1.59, 95% CI 1.13–2.22). Finally, chest drain strategy was associated with PPC risk: one-sided plus Jackson-Pratt drain was protective (OR 0.48, 95% CI 0.25–0.91), whereas bilateral drains markedly increased risk (OR 2.73, 95% CI 1.45–5.15), compared with a single one-sided drain. The predictive model demonstrated modest discrimination, with a pooled AUC of 0.616 (95% CI 0.575–0.657 (Table 5)).

**Table 4:** Independent predictors of postoperative pulmonary complications

**Variable**	**OR**	**95% CI**	***P*-value**
**Smoking**			
Non-smoker	Ref		
Former smoker	1.454	(1.027–2.059)	0.035
Smoker	1.742	(1.154–2.631)	0.008
**Drain type**			
One-sided (right or left)	Ref		
One-sided + JP drain (right or left + JP)	0.479	(0.251–0.912)	0.025
Bilateral	2.592	(1.380–4.870)	0.003
**Analgesia type**			
Epidural	Ref		
Paravertebral	1.498	(1.033–2.172)	0.033
Other^a^	1.574	(0.879-2.817)	0.127
**Location of anastomosis**			
Intrathoracic	Ref		
Cervical	1.636	(1.169–2.291)	0.004

Abbreviations: CI, confidence interval; OR, odds ratio; JP, Jackson-Pratt.

^a^Medication (such as: sufentanil pump and/or *esketamine*).

**Table 5:** Assessment of the prediction model

	**AUC (95% CI)**	**R** ^**2**^
Pooled dataset	0.616 (0.575–0.657)	0.060
Internal validated dataset	0.598	0.038

Abbreviations: AUC, area under the curve; CI, confidence interval; R^2^, coefficient of determination.

A sensitivity analysis including an interaction term between drain strategy and date of surgery showed no statistically significant effect (OR 1.00, 95% CI 1.00–1.00, *P* = 0.417), and the interaction was not retained in the final model.

After internal validation, the AUC was 0.598. The explained variance (Nagelkerke R^2^) was 0.067 before validation and 0.038 after validation. The shrinkage factor was 0.872. The calibration curve showed good agreement between predicted and observed probabilities, with minimal deviation from the reference line (Supplementary Fig. 1). Predicted risks, however, did not exceed 0.50 (Supplementary Table 2).

Because postoperative pneumonia (PP) represents the most frequent component of PPCs, we performed an exploratory subanalysis using PP as the isolated outcome. Pneumonia was defined by clinical symptoms with radiographic confirmation (n = 126), and severe cases were classified as Clavien–Dindo grade ≥ III (n = 21) by registered physicians. However, the limited number of events resulted in unstable variable selection and insufficient model robustness. Reduced models demonstrated selection instability, including loss of anastomotic location, and the slightly higher AUC observed reflected overfitting rather than improved performance. Consequently, the pneumonia-only model was not retained, supporting use of the composite ECCG PPC definition in the final model. (Supplementary Tables 3 and 4) An exploratory point-based scoring system is presented in Supplementary Table 5.

## DISCUSSION

This single-center study from the Netherlands identified pre- and perioperative risk factors for pulmonary complications following esophagectomy. Smoking status, chest drain strategy, analgesia type, and anastomotic location emerged as independent predictors. These results suggest that preoperative counseling should emphasize modifiable lifestyle factors, particularly smoking cessation, while perioperative management choices may inform postoperative monitoring strategies.

Smoking was a strong risk factor for PPCs, underscoring the importance of cessation before surgery. Evidence from thoracic surgery indicates that at least 4 weeks of abstinence can reduce the risk of PPCs, without a paradoxical increase among patients who quit shortly before surgery.^27^ Thus, smoking cessation can be safely encouraged at any stage prior to esophagectomy.^28^ While prior studies have identified smoking, BMI, and surgical approach as PPC risk factors,^8^^,^^15^^,^^29^ this study adds new insights by incorporating underexplored perioperative variables. Namely drain type, analgesia method, and location of anastomosis. These factors are rarely included in existing models but may carry significant clinical relevance for perioperative decision-making.

Notably, the use of Jackson-Pratt drains was associated with a lower incidence of PPCs, suggesting a potential benefit of this drainage method. Although causality cannot be established in this retrospective analysis, a plausible mechanism is that improved drainage reduces pleural fluid accumulation with subsequent pulmonary atelectasis, and thereby lower pulmonary risk. As the JP drain was introduced in 2021, a sensitivity analysis tested for time-related bias; the interaction with year of surgery was not significant (OR 1.00, 95% CI 1.00–1.00, *P* = 0.417), indicating the association was not confounded by temporal change. These findings warrant confirmation in larger observational or multicenter datasets.

Our results further highlight the potential impact of perioperative management choices. ERAS protocols were implemented prior to the study period and applied consistently, precluding evaluation of their independent effect, although their benefit in reducing postoperative morbidity is well established.^30^ Cervical anastomosis was associated with higher PPC risk compared with intrathoracic anastomosis. This association is unlikely to reflect a direct causal effect of anastomotic location alone.^19^ The higher anastomotic leak rate associated with cervical reconstruction may contribute to secondary pulmonary complications through infection and systemic inflammatory responses. In our cohort, these findings therefore support increased perioperative awareness rather than a change in surgical strategy. Moreover, non-epidural analgesia showed a signal toward increased complications. Although previous randomized trials, including our own PEPMEN trial, demonstrated equivalent analgesic efficacy of epidural and paravertebral techniques,^21^ our observational analysis suggested higher PPC rates with non-epidural regimens. Given the likelihood of residual confounding and practice variability, these findings should be interpreted cautiously and not as evidence of superiority of one technique. Although previous studies have reported an association between the extent of lymphadenectomy and pulmonary complications, in our cohort lymphadenectomy was largely standardized and closely related to surgical approach and anastomotic location, which may limit its independent contribution to risk prediction.^31^

Several aspects of this study strengthen the validity of the findings. Multiple imputation was applied to address missing data. Incorporating neoadjuvant therapy helped adjust for treatment-related confounding, a factor often omitted in earlier models. Despite these strengths, limitations include limited granularity of smoking data (no pack-years or cessation duration), reliance on clinically diagnosed PPCs with possible interobserver variability, and methodological adaptations for BMI and pulmonary function measures.

Although previous studies have identified age as risk factor for PPCs,^9^ this variable was not independently associated in our model. This may reflect the relatively homogeneous and preselected surgical population, as well as attenuation of their effects in multivariable analysis.

Consistent with prior studies,^16^^,^^32^ FEV1/VC was not an independent predictor in our model, suggesting limited discriminatory value of basic spirometry alone. More dynamic measures, such as gas exchange, intraoperative ventilation pressures, or hemodynamic indices, may better capture pulmonary risk and warrant evaluation in future models.^33^^,^^34^ In addition, detailed intraoperative variables such as ventilation strategy, operative time, and intraoperative blood loss were not consistently available for all patients. Although surgical procedures were standardized and operative times were comparable across the cohort, inclusion of these variables may further improve predictive performance in future models.

In addition, surgical approach and type of resection were included in the analysis, thereby accounting for the presence of thoracotomy. Given the predominance of minimally invasive procedures in our cohort, variability in thoracotomy was limited. Moreover, drain configuration was consistent across surgical approaches, suggesting that its association with pulmonary complications is not solely explained by the presence of thoracotomy. Furthermore, the ECCG definition includes procedure-related events such as tracheobronchial fistula and prolonged air leak; their inclusion enables comparison across studies but introduces heterogeneity. Variable selection was restricted to clinically relevant and consistently available factors. Inclusion of additional variables does not necessarily improve predictive performance and may increase the risk of overfitting, particularly in relation to the number of outcome events. From a methodological perspective, the cohort size (n = 960) was just below the predefined sample size target of 965, yet the large number of outcome events (n = 254) comfortably exceeded accepted thresholds for model stability. Finally, although PPCs represent a heterogeneous group of complications, an exploratory subgroup analysis separating infectious–inflammatory and mechanical events resulted in lower model performance. Therefore, consistent with previous ECCG-based studies, the composite PPC definition was retained.

The identification of modifiable factors such as smoking highlights the role of prehabilitation. High-risk patients, particularly smokers or those receiving non-epidural analgesia, may benefit from tailored perioperative strategies and enhanced monitoring. The potential role of JP drains also merits further exploration, including their association with outcomes beyond PPCs, such as anastomotic healing or recovery time.

The predictive model achieved an AUC of 0.60, slightly lower than but broadly comparable to prior PPC models (0.64–0.71).^9^^,^^17^ Although the model demonstrated good calibration, its discriminative performance was limited, indicating that while predicted risks reflect observed event rates, the ability to separate high- from low-risk cases remains poor. This modest performance reflects the multifactorial and heterogeneous nature of PPCs.

From a clinical perspective, the current model should therefore be interpreted as a framework for risk estimation rather than a tool for individual decision-making. An exploratory point-based scoring system was derived as a sensitivity analysis and is provided in the Supplementary Materials. Further refinement and external validation are required before translation into a simplified clinical risk score.

To enhance predictive accuracy, future models should incorporate variables such as inflammatory biomarkers, respiratory muscle strength, and intraoperative blood loss and ventilation parameters. Machine learning approaches may also help improve discrimination and uncover non-linear patterns missed by traditional methods.

In conclusion, this study identified smoking, bilateral drains, paravertebral analgesia, and cervical anastomosis as independent predictors of pulmonary complications after esophagectomy. These findings support ongoing changes in less invasive drain practice and emphasize targeted perioperative strategies, including structured smoking cessation programs, optimization of analgesia, and tailored postoperative monitoring. Although the predictive performance of the model was modest, it provides a clinically relevant framework for risk assessment. Future studies should focus on integrating more detailed physiological and intraoperative variables and on external validation to improve predictive accuracy and enable development of robust risk stratification tools in esophageal surgery.

## Supplementary Material

doag041_Supplemental_Files

## Notes

Specific author contributions: DvdA and RK contributed equally to this manuscript and therefore share first authorship. Conceptualization, DvdA, RK, MvBH; methodology, DvdA, RK, SSG; formal analysis, data curation, RK, DvdA; draft preparation, DvdA, RK; review and editing, MvBH, FD, WE, SSG; supervision, MvBH, WE, FD, SSG. All authors have read and agreed to the published version of the manuscript.

Financial support: This study received no funding.

Declaration of interest: SSG is a consultant for Medicaroid, Olympus and J&J. MvBH reports consulting or advisory roles for Viatris, Johnson & Johnson, BBraun, Stryker, and Medtronic.
