# Supplementary material for: Postoperative pulmonary complications after esophagectomy: risk factors and prediction model
Source: Dis Esophagus. 2026 Apr 21;39(2):doag041. doi: 10.1093/dote/doag041 (PMC13096804; doi:10.1093/dote/doag041)
Supplement: doag041_Supplemental_Files [file doag041_supplemental_files.zip › SUPPLEMENTAL_FILE_1_doag041.docx]

**SUPPLEMENTAL FILE 1**

| **Supplementary Table 1.** Definitions of Postoperative Pulmonary Complications according to the ECCG | |
| --- | --- |
| **Complication** | **Definition** |
| Pneumonia | New or progressive pulmonary infiltrate on chest imaging, in combination with clinical signs (e.g., fever, leukocytosis, purulent sputum, hypoxemia), and when available microbiological confirmation (ATS/IDSA criteria). |
| Pleural effusion | Pleural fluid collection requiring an additional drainage procedure. |
| Pneumothorax | Pneumothorax requiring active treatment (needle aspiration, chest tube insertion, or surgical intervention). |
| Atelectasis | Lobar or segmental collapse due to mucus plugging requiring bronchoscopy. |
| Respiratory failure | Inability to maintain adequate oxygenation or ventilation necessitating reintubation. |
| Acute aspiration | Documented aspiration event leading to respiratory compromise requiring medical intervention. |
| ARDS | Acute respiratory distress syndrome defined by the Berlin criteria(24) (acute onset, bilateral opacities, non-cardiac cause, impaired oxygenation). |
| Tracheobronchial injury | Intraoperative or postoperative injury to the trachea or bronchi confirmed clinically or radiologically. |
| Persistent air leakage | Air leak necessitating chest tube maintenance beyond 10 postoperative days. |
| *Abbreviations: ECCG = Esophagectomy Complications Consensus Group* (22) | |

| **Supplementary Table 2.** Adjusted regression coefficients and intercept | |
| --- | --- |
| **Variable** | **Estimate** |
| **Intercept** | -1.480 |
| **Smoking** |  |
| Non-smoker | Ref |
| Former smoker | 0.317 |
| Smoker | 0.470 |
| **Drain type** |  |
| One-sided (right or left) | Ref |
| One-sided + JP drain (right or left + JP) | -0.623 |
| Bilateral | 0.806 |
| **Analgesia type** |  |
| Epidural | Ref |
| Paravertebral | 0.342 |
| Other^1^ | 0.384 |
| **Location of anastomosis** |  |
| Intrathoracic | Ref |
| Cervical | 0.417 |
| *Abbreviations: JP= Jackson-Pratt*  ^1^: medication (such as: sufentanil pump and/or *esketamine*) | |

| **Supplementary Figure 1.** Calibration plot (reliability curve) |
| --- |
|  |

| **Supplementary Table 3.** Risk factors for postoperative pneumonia | | | |
| --- | --- | --- | --- |
|  | **Univariable analysis** | | |
| **Variable** | **OR** | **95% CI** | **p-value** |
| **Sex** |  |  |  |
| Male | Ref |  |  |
| Female | 1.354 | (0.519-3.532) | 0.535 |
| Age (in years) | 1.017 | (0.966-1.070) | 0.533 |
| **BMI** (in Kg/m^2^) |  |  |  |
| ≤ 30 |  |  |  |
| >30 | 1.900 | (0.685-5.273) | 0.218 |
| FEV1VC^1^ (in %) | 0.966 | (0.926-1.007) | 0.104 |
| **FEV1VC** (in %) |  |  |  |
| >70 |  |  |  |
| ≤70 | 1.871 | (0.767-4.561) | 0.169 |
| **ASA-score** |  |  |  |
| I | Ref |  |  |
| II | 1.141 | (0.315-4.136) | 0.841 |
| III/IV | 1.778 | (0.453-6.976) | 0.409 |
| **Smoking** |  |  |  |
| Non-smoker | Ref |  |  |
| Former smoker | 1.819 | (0.565-5.850) | 0.316 |
| Smoker | 2.991 | (0.864-10.351) | 0.084 |
| **Diabetes mellitus** |  |  |  |
| No |  |  |  |
| Yes | 2.428 | (0.872-6.760) | 0.089 |
| **Chronic lung disease (COPD/ Asthma)** |  |  |  |
| No | Ref |  |  |
| Yes | 1.958 | (0.564-6.798) | 0.290 |
| **Neo-adjuvant therapy** |  |  |  |
| No neoadjuvant therapy | Ref |  |  |
| Chemotherapy | 0.000 | (0.000-) | 0.997 |
| Chemoradiotherapy | 0.993 | (0.227-4.335) | 0.992 |
| **Histology** |  |  |  |
| Adenocarcinoma | Ref |  |  |
| Squamous cell carcinoma | 0.762 | (0.221-2.630) | 0.668 |
| Other histology^2^ | 0.228 | (0.228-13.898) | 0.583 |
| **Approach surgery** |  |  |  |
| Open | Ref |  |  |
| Hybrid | 0.460 | (0.089-2.379) | 0.354 |
| MIE | 0.241 | (0.067-0.867) | **0.029** |
| **Salvage** |  |  |  |
| No | Ref |  |  |
| Yes | 5.120 | (1.650-15.887) | **0.005** |
| **Drain type** |  |  |  |
| One-sided | Ref |  |  |
| One-sided double | 0.608 | (0.080-4.618) | 0.631 |
| Both | 1.926 | (0.434-8.542) | 0.388 |
| **Drain type^3^** |  |  |  |
| Both sided | Ref |  |  |
| One-sided | 0.519 | (0.324-0.832) | **0.006** |
| One-sided double | 0.316 | (0.147-0.680) | **0.003** |
| **Anastomosis location** |  |  |  |
| Intrathoracic | Ref |  |  |
| Neck | 0.597 | (0.171-2.082) | 0.419 |
| None | 7.389 | (1.973-27.681) | **0.003** |
| **Analgesia type** |  |  |  |
| Epidural | Ref |  |  |
| Paravertebral | 1.148 | (0.373-3.529) | 0.810 |
| Other^4^ | 2.656 | (0.742-9.510) | 0.133 |
| Abbreviations: FEV1= Forced expiratory volume in 1 second; BMI= body mass index; ASA-score: American society of Anesthesiologists Physical status; COPD=chronic obstructive pulmonary disease; MIE = minimally invasive esophagectomy (MIE Thoracic/ MIE Abdominal); JP= Jackson-Pratt; *: not used in the multivariable analysis; ^1^: pooled in the imputed dataset;^2^: : signet ring cell-, poorly differentiated-, neuroendocrine, neuro-squamous carcinoma;^3^: different reference category;^4^: medication (such as: sufentamil pump and/or esklemaine) | | | |

| **Supplementary Table 4.** Assessment of the prediction model | | |
| --- | --- | --- |
|  | **AUC (95% CI)** | **R^2^** |
| Pooled dataset | 0.643 (0.626-0.661) | 0.058 |
| Abbreviations: AUC; area under the curve, CI; confidence interval, R^2^; coefficient of determination | | |

| **Supplementary Table 5.** Multivariable prediction model for postoperative pulmonary complications | | | |
| --- | --- | --- | --- |
| **Predictor** | **Variable** | **β** | **OR (95% CI)** |
| Smoking | Former | 0.317 | 1.454 (1.027–2.059) |
| Smoking | Smoker | 0.470 | 1.742 (1.154–2.631) |
| Drain type | One-sided + JP | -0.623 | 0.479 (0.251–0.912) |
| Drain type | Bilateral | 0.806 | 2.592 (1.380–4.870) |
| Analgesia | Paravertebral | 0.342 | 1.498 (1.033–2.172) |
| Analgesia | Other | 0.384 | 1.574 (0.879–2.817) |
| Anastomosis | Cervical | 0.417 | 1.636 (1.169–2.291) |
| Intercept | - | -1.480 | - |
| Abbreviations: JP= Jackson-Pratt | | | |

| **Supplementary Table 6.** Exploratory point-based risk score derived from the multivariable model | |
| --- | --- |
| **Risk Factor** | **Points** |
| Smoking – Former | +1 |
| Smoking – Current | +2 |
| Drain type – One-sided + JP | -2 |
| Drain type – Bilateral | +3 |
| Analgesia – Paravertebral | +1 |
| Analgesia – Other | +1 |
| Anastomosis – Cervical | +1 |
| Abbreviations: JP= Jackson-Pratt | |

| **Supplementary Table 7.** Drain configuration | | | | |
| --- | --- | --- | --- | --- |
|  | **One sided drain (left or right)**  **N (%)** | **One sided + JP drain**  **N (%)** | **Bilateral drains**  **N (%)** | **Total** |
| **Open surgery** | 27 (77.1) | 2 (5.7) | 6 (17.1) | 35 |
| **Hybrid** | 69 (81.2) | 4 (4.7) | 12 (14.1) | 85 |
| **Minimally invasive** | 741 (88.2) | 71 (8.5) | 28 (3.3) | 840 |
| **Total** | 837 (87.2) | 77 (8.0) | 46 (4.8) | 960 |
| Abbreviations: JP= Jackson-Pratt | | | | |
