# Supplementary figures and images for: Postoperative pulmonary complications after esophagectomy: risk factors and prediction model
Source: Dis Esophagus. 2026 Apr 21;39(2):doag041. doi: 10.1093/dote/doag041 (PMC13096804; doi:10.1093/dote/doag041)

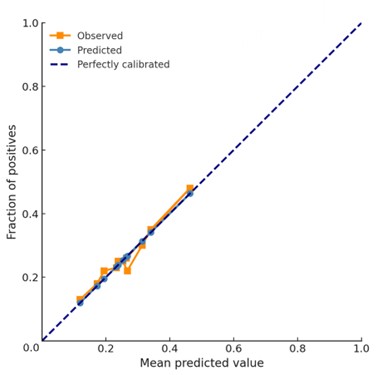

Supplement: doag041_Supplemental_Files [file doag041_supplemental_files.zip › SuppFigure1Calibrationplot_doag041.jpg]
